# Tanshinone IIA restores CD8^+^ T-cell immunity in TNBC by suppressing the IDO1–kynurenine axis

**DOI:** 10.3389/fphar.2026.1903253

**Published:** 2026-08-25

**Authors:** Zhu Qiu, Chao Meng, Maocai Tang

**Affiliations:** 1 Department of Breast and Thyroid Surgery, Chongqing Key Laboratory of Molecular Oncology and Epigenetics, The First Affiliated Hospital of Chongqing Medical University, Chongqing, China; 2 Chongqing Xianxin Medical Technology Co., Ltd., Chongqing, China; 3 School of Biomedical Engineering, Chongqing Medical University, Chongqing, China; 4 Department of Gastrointestinal Surgery, Chongqing University Cancer Hospital, Chongqing, China

**Keywords:** indoleamine 2,3-dioxygenase 1, tryptophan 2,3-dioxygenase 2, immune checkpoint blockade resistance, kynurenine, tanshinone IIA, triple-negative breast cancer

## Abstract

**Introduction:**

Immune checkpoint blockade (ICB) benefits only a minority of patients with triple-negative breast cancer (TNBC), partly because tryptophan catabolism through indoleamine 2,3-dioxygenase 1 (IDO1) and tryptophan 2,3-dioxygenase 2 (TDO2) generates kynurenine, activates aryl hydrocarbon receptor (AhR) signaling, and suppresses CD8^+^ T-cell function. We investigated whether tanshinone IIA (Tan-IIA) modulates this pathway.

**Methods:**

We integrated network pharmacology with TCGA Breast Invasive Carcinoma transcriptomic analyses and evaluated Tan-IIA in 4T1 and MDA-MB-231 cells, tumor-conditioned-medium CD8^+^ T-cell assays, kynurenine add-back and IDO1-overexpression rescue experiments, and syngeneic 4T1 and EMT6 tumor models.

**Results:**

IDO1 and TDO2 were identified as upregulated Tan-IIA-associated candidates linked to an inflamed-yet-dysfunctional immune phenotype. Tan-IIA dose-dependently suppressed basal and IFN-γ-induced IDO1 and TDO2 expression, reduced the Kyn/Trp ratio and IDO activity, downregulated AhR target genes in CD8^+^ T cells, and restored CD8^+^ T-cell proliferation and effector-marker expression. Exogenous kynurenine and Ido1 overexpression substantially reversed these effects. In vivo, Tan-IIA reduced tumor growth, lowered tumor IDO1 and TDO2 expression, enhanced T-cell infiltration, and showed CD8-dependent antitumor activity. In checkpoint-resistant 4T1 tumors, Tan-IIA combined with anti-PD-1 produced the strongest tumor control and longest survival.

**Discussion:**

Tan-IIA relieves IDO1/TDO2–kynurenine–AhR metabolic immunosuppression and sensitizes TNBC to checkpoint blockade, supporting its further evaluation as a microenvironment-remodeling partner for ICB-resistant TNBC.

## 1 Introduction

Triple-negative breast cancer (TNBC) lacks estrogen receptor, progesterone receptor, and HER2 expression and remains the breast cancer subtype with the fewest targeted options and the poorest outcomes. Because TNBC carries a relatively high mutational and immune burden, immune checkpoint blockade (ICB) entered clinical practice through trials such as IMpassion130 and KEYNOTE-355, in which anti-PD-L1 or anti-PD-1 added to chemotherapy improved outcomes in PD-L1-positive metastatic disease (Schmid et al., 2018; Cortes et al., 2020). However, durable benefit is confined to a minority of patients, and both primary and acquired resistance are common. Many TNBCs exhibit a suppressive immune microenvironment in which cytotoxic T cells are numerically limited, spatially restrained, or functionally exhausted by myeloid cells, regulatory T cells, and metabolic checkpoints (Ji et al., 2018). The density of tumor-infiltrating lymphocytes is prognostic in TNBC (Denkert et al., 2018), so restoring T-cell infiltration and function remains a priority for extending the reach of ICB.

One conserved mechanism of immune suppression is metabolic. The essential amino acid tryptophan is catabolized along the kynurenine pathway by IDO1 and TDO2, depleting local tryptophan and accumulating kynurenine (Platten et al., 2019). Kynurenine is an endogenous ligand of the aryl hydrocarbon receptor (AhR); upon binding, AhR drives transcriptional programs that impair effector T cells, promote regulatory T-cell differentiation, and support tolerogenic myeloid cells (Opitz et al., 2011; Mezrich et al., 2010; Gandhi et al., 2010). In tumors, IDO1 is frequently induced by IFN-γ released from infiltrating T cells, creating an adaptive feedback loop in which the immune response provokes its own metabolic suppression (Munn and Mellor, 2016; Spranger et al., 2013; Bessede et al., 2014). Kynurenine, taken up by CD8^+^ T cells, activates AhR and upregulates PD-1, directly linking tryptophan catabolism to checkpoint biology (Liu et al., 2018; Campesato et al., 2020). Clinically, the failure of the IDO1 inhibitor epacadostat to improve outcomes when added to pembrolizumab (Long et al., 2019) suggests that selective single-enzyme blockade may be insufficient and that broader modulation of the IDO1/TDO2–kynurenine–AhR axis warrants investigation (Prendergast et al., 2017).

Tanshinone IIA (Tan-IIA) is a lipophilic diterpenoid obtained from the root of *Salvia miltiorrhiza* (Danshen) with reported anti-tumor activity, including inhibition of proliferation, migration, and stemness in breast cancer models (Wang et al., 2005; Fang et al., 2021; He et al., 2023; Lin et al., 2013). AhR signaling has also been linked to breast cancer inflammation and progression (Guarnieri, 2020), and kynurenine-pathway metabolites engage oncogenic signaling beyond immunity (Bishnupuri et al., 2019). Whether Tan-IIA acts on tumor tryptophan metabolism and thereby restores CD8^+^ T-cell function in TNBC has not been tested. In this study, we combined network pharmacology and TCGA Breast Invasive Carcinoma (TCGA–BRCA) transcriptomics to nominate IDO1 and TDO2 as Tan-IIA-associated candidate genes enriched within the kynurenine pathway and then evaluated the hypothesis experimentally. We show that Tan-IIA suppresses IDO1/TDO2 expression and activity, lowers the Kyn/Trp ratio, reduces AhR target genes, restores CD8^+^ T-cell function in a kynurenine- and IDO1-dependent manner, and sensitizes checkpoint-resistant 4T1 tumors to anti-PD-1 therapy.

## 2 Materials and methods

### 2.1 Bioinformatics analysis

Putative Tan-IIA-associated protein targets were retrieved from SwissTargetPrediction (http://www.swisstargetprediction.ch/) and TCMSP (https://old.tcmsp-e.com/) using the canonical Tan-IIA structure (PubChem CID 164676; CAS 568-72-9) and standardized to official gene symbols. RNA-seq expression data and clinical annotation for the TCGA–BRCA cohort were obtained from the GDC portal. TNBC cases (n = 171), defined by negative ER/PR/HER2 status, were compared with adjacent normal tissue (n = 113). Differential expression was performed using edgeR and limma-voom. Lowly expressed genes were filtered using filterByExpr, library sizes were normalized using the trimmed mean of M values (TMM), and differential expression was tested using limma with empirical Bayes moderation. Genes with |log_2_ fold change| > 1 and Benjamini–Hochberg-adjusted *p* < 0.05 were considered differentially expressed. Tan-IIA-associated targets were intersected with TNBC up- and downregulated genes, and the 43 overlapping genes were subjected to Gene Ontology and KEGG enrichment using clusterProfiler (Wu et al., 2021). The 11 upregulated overlapping genes are listed in Supplementary Tables S1 and S2. Immune cell composition was estimated using CIBERSORT (Newman et al., 2015), and T-cell dysfunction and exclusion scores were obtained using TIDE (Ji et al., 2018). An *IDO1*/*TDO2* score was defined as the mean z-scored expression of IDO1 and TDO2. Correlations used Pearson coefficients; two-group comparisons used Wilcoxon rank-sum tests unless otherwise stated. For the IDO1/TDO2 composite score, *IDO1* and *TDO2* logCPM values in TCGA–BRCA TNBC samples were converted to z-scores separately, and the mean of the two z-scored values was used as the pathway score for each sample.

### 2.2 Compounds and key reagents

Tan-IIA was dissolved in DMSO for *in vitro* use (final DMSO <0.1%). For animal studies, Tan-IIA was formulated in 5% DMSO/40% PEG300/5% Tween-80/50% saline or another validated vehicle compatible with intraperitoneal injection. Key compounds, antibodies, and functional reagents are listed in Table 1.

**TABLE 1:** Key compounds, antibodies, and functional reagents.

Reagent/antibody	Working concentration/dilution	Supplier (Cat#)
Tanshinone IIA	0–80 µM *in vitro*; 40 mg/kg, i.p., *in vivo*	MCE (HY-N0135)
Recombinant murine IFN-γ	20 ng/mL	Thermo Fisher (315-05)
Recombinant human IFN-γ	20 ng/mL	Thermo Fisher (300-02)
L-kynurenine	100 µM for rescue	Sigma-Aldrich (K8625)
Kynurenine/tryptophan ratio ELISA pack	According to the manufacturer’s protocol	ImmuSmol (ISE-2227R)
L-tryptophan	200 µM	Sigma-Aldrich (T0254)
L-ascorbic acid	20 mM	Sigma-Aldrich (A92902)
Methylene blue	10 µM	Sigma-Aldrich (M9140)
Catalase	100 μg/mL	Sigma-Aldrich (C9322)
Trichloroacetic acid	10%	Sigma-Aldrich (T6399)
4-(Dimethylamino)benzaldehyde/Ehrlich’s reagent	2% w/v in glacial acetic acid	Sigma-Aldrich (156477)
Anti-mouse PD-1, clone RMP1-14	200 µg/mouse, i.p., q3d	Bio X cell (BE0146)
Rat IgG2a isotype control, clone 2A3	200 µg/mouse, i.p	Bio X cell (BE0089)
Anti-mouse CD8α depleting antibody, clone 2.43	200 µg/mouse, i.p	Bio X cell (BE0061)
Fixable viability stain 450	1:1000	BD Biosciences (562247)
Anti-mouse CD16/32, clone 93	1 µg/10^6^ cells	BioLegend (101302)
Anti-mouse CD45-Alexa Fluor 700, clone 30-F11	1:100	BioLegend (103128)
Anti-mouse CD8a-PE/Cy7, clone 53-6.7	1:100	BioLegend (100722)
Anti-mouse CD4-PerCP/Cy5.5, clone GK1.5	1:100	BioLegend (100434)
Anti-mouse FoxP3-Alexa Fluor 488, clone MF-14	1:100	BioLegend (126406)
Anti-mouse CD11b-Brilliant Violet 510, clone M1/70	1:100	BioLegend (101245)
Anti-mouse F4/80-Brilliant Violet 605, clone BM8	1:100	BioLegend (123133)
Anti-mouse Ly6G/Ly6C (Gr-1)-PE, clone RB6-8C5	1:100	BioLegend (108408)
Anti-mouse CD3-PerCP/Cy5.5, clone 17A2	1:100	BioLegend (100218)
Anti-mouse IFN-γ-APC, clone XMG1.2	1:100	BioLegend (505810)
Anti-human/mouse granzyme B-PE, clone QA16A02	1:100	BioLegend (372208)
Anti-mouse Perforin-PE, clone S16009A	1:100	BioLegend (154306)
Anti-mouse Ki-67-Brilliant Violet 605, clone 16A8	1:100	BioLegend (652413)
Anti-mouse IDO1-Alexa Fluor 647, clone 2E2/IDO1	1:100	BioLegend (654004)
Anti-human IDO1-PE, clone W20099D	1:100	BioLegend (335854)
Anti-human TDO2-Alexa Fluor 700, clone 998604	1 µg/10^6^ cells	R&D systems (IC9768N)
anti-mouse TDO2 antibody	2 µg/1 × 10^6^ cells	Boster Bio/ABMIUM (A07526-2)
Goat anti-rabbit IgG (H + L) cross-adsorbed secondary antibody, Alexa Fluor 647	4 μg/mL	Thermo Fisher (A-21244)
Foxp3/transcription factor staining buffer set	According to the manufacturer’s protocol	Thermo Fisher (00-5523–00)
Anti-IDO1 antibody [EPR28349-89]	1:200	Abcam (ab311847)
Anti-TDO2/TDO antibody [EPR29352-720]	1:200	Abcam (ab325323)
Anti-CD8 alpha antibody [EPR20305]	1:200	Abcam (ab209775)
PV-9000 universal two-step IHC detection kit	According to the manufacturer’s protocol	Zhongshan Golden Bridge (PV-9000)
Anti-CD3 epsilon antibody [SP7]	1:200	Abcam (ab16669)
Anti-alpha smooth muscle actin antibody [1A4]	1:200	Abcam (ab7817)
Goat anti-rabbit IgG (H + L), Alexa Fluor 488	1:500	Invitrogen (A-11008)
Goat anti-mouse IgG (H + L), Alexa Fluor 594	1:500	Invitrogen (A-11005)
ProLong Gold Antifade Mountant with DAPI	10 µL/slide	Thermo Fisher (P36931)

### 2.3 Cell culture

Murine 4T1 and EMT6 mammary carcinoma cells and human MDA-MB-231 TNBC cells were maintained in RPMI-1640 or DMEM supplemented with 10% fetal bovine serum and 1% penicillin–streptomycin at 37 °C in 5% CO_2_. Cell lines were authenticated by short tandem repeat profiling, where applicable, and confirmed to be mycoplasma-negative before use.

### 2.4 Cell viability and IC_50_ determination

Cells were seeded in 96-well plates and treated with Tan-IIA across eight doses (0, 1.25, 2.5, 5, 10, 20, 40, and 80 µM). Viability was measured using the CCK-8 assay at 72 h; time-course experiments sampled selected doses at 0–96 h. Absorbance at 450 nm was measured using a microplate reader. IC_50_ values were derived by nonlinear regression (log [inhibitor] vs. normalized response, variable slope) using GraphPad Prism. Each condition was tested in technical triplicate across three independent biological experiments (n = 3).

### 2.5 RT-qPCR and intracellular flow cytometry of IDO1/TDO2

To model an inflamed microenvironment, tumor cells were treated with species-matched IFN-γ (20 ng/mL) and graded Tan-IIA (0–20 µM) for 24–48 h. Total RNA was extracted using TRIzol or an equivalent column-based RNA extraction kit according to the manufacturer’s instructions. RNA concentration and purity were assessed by spectrophotometry, and samples with A260/280 values of approximately 1.8–2.1 and no evident degradation were used for reverse transcription. *IDO1/Ido1*, *TDO2/Tdo2*, and AhR-target transcripts were quantified using the 2^−ΔΔCt^ method with *GAPDH/Gapdh* or *ACTB/Actb* as reference genes. Primer sequences are provided in Supplementary Table S5. For intracellular flow cytometry, cells were stained with fixable viability dye, fixed and permeabilized using a FoxP3/transcription factor-compatible fixation/permeabilization buffer or equivalent, and then stained with the antibodies listed in Table 1. IDO1 was detected using directly conjugated intracellular flow-cytometry antibodies. Human TDO2 was detected using directly conjugated anti-human TDO2-Alexa Fluor 700. For mouse TDO2, an in-house optimized indirect intracellular staining strategy was used with an unconjugated rabbit anti-TDO2 primary antibody, followed by a flow-compatible Alexa Fluor 647-conjugated goat anti-rabbit IgG secondary antibody. The results were reported as median fluorescence intensity (MFI) after exclusion of debris, doublets, and dead cells.

### 2.6 Tumor-CD8^+^ T-cell co-culture and functional assays

Tumor cells were treated with vehicle or Tan-IIA for 72 h, and conditioned medium (Control-CM, Tan-IIA-CM, or Tan-IIA-CM supplemented with 100 µM kynurenine) was collected. Mouse CD8^+^ T cells were isolated from the spleen, activated with anti-CD3/CD28, labeled with CFSE, and cultured in each conditioned medium. Proliferation was assessed using CFSE dilution. For effector-function analysis, cells were restimulated in the presence of brefeldin A and monensin and stained for intracellular IFN-γ and granzyme B. AhR target genes (Cyp1a1, Cyp1b1, and Ahrr) were measured through RT-qPCR. For causality, parallel co-cultures used tumor cells transfected with an IDO1 overexpression plasmid or empty vector before Tan-IIA treatment; IDO1 overexpression was verified through RT-qPCR and immunoblotting before conditioned medium was collected.

For Kyn/Trp analysis, tumor-cell-conditioned medium was collected after 72 h of treatment and centrifuged at 2,000 × g for 10 min at 4 °C to remove cells and debris. The cell-free supernatant was aliquoted and stored at −80 °C until measurement. Kynurenine and tryptophan concentrations were quantified from the same conditioned-medium samples using a kynurenine/tryptophan ratio ELISA pack (ImmuSmol, ISE-2227R), which includes separate competitive ELISA assays for L-kynurenine and L-tryptophan. Samples, standards, and controls were processed according to the manufacturer’s protocol, and absorbance was measured at 450 nm with wavelength correction at 620–650 nm using a microplate reader. The Kyn/Trp ratio was calculated as kynurenine concentration divided by tryptophan concentration and reported as Kyn/Trp × 10^3^.

IDO enzymatic activity was measured using a manual colorimetric kynurenine-production assay. In brief, tumor-cell lysates were normalized by total protein concentration and incubated at 37 °C for 60 min in reaction buffer containing 50 mM potassium phosphate buffer (pH 6.5), 200 μM L-tryptophan, 20 mM L-ascorbic acid, 10 µM methylene blue, and 100 μg/mL catalase. The reaction was terminated by adding trichloroacetic acid to a final concentration of 10% and heating at 65 °C for 15 min to convert N-formylkynurenine to kynurenine. After centrifugation at 12,000 × g for 10 min, the clarified supernatant was mixed with an equal volume of Ehrlich’s reagent, prepared as 2% 4-dimethylaminobenzaldehyde in glacial acetic acid. Absorbance was measured at 480 nm using a microplate reader. Kynurenine generated during the reaction was calculated from an L-kynurenine standard curve, and IDO activity was reported as pmol kynurenine generated per min per µg protein. IDO1 overexpression was confirmed by RT-qPCR and immunoblotting before the rescue experiments, and the validation results are provided in Supplementary Figures S2A,B. Representative flow-cytometry plots were gated on live singlet CD8^+^ T cells, and IFN-γ^+^ or GZMB^+^ values indicate the percentage of cytokine-positive cells within the CD8^+^ parent gate.

### 2.7 Animal studies

Female BALB/c mice (6–8 weeks) were inoculated orthotopically with 4T1 cells in the mammary fat pad. Where indicated, EMT6 cells were used as an additional syngeneic mammary carcinoma model. After randomization, when tumors reached approximately 60–80 mm^3^, animals received vehicle, Tan-IIA (40 mg/kg, i. p., twice weekly), anti-PD-1 antibody (clone RMP1-14, 200 µg/mouse, i. p., every 3 days), or the Tan-IIA plus anti-PD-1 combination (n = 5 per group unless otherwise stated). Tumor volume was calculated as 0.5 × length × width^2^, and body weight was recorded on the same days as tumor volume measurements during treatment as an indicator of systemic tolerability. Endpoint tumors were weighed, dissociated for immune profiling by flow cytometry, and processed for immunohistochemistry or multiplex immunofluorescence. Survival was followed to humane endpoint criteria. All procedures were approved by the Institutional Animal Care and Use Committee (approval no. 2517002 to be confirmed) and complied with ARRIVE guidelines.

For tumor immune profiling, single-cell suspensions were first gated on cells, singlets, live cells, and CD45^+^ leukocytes. Flow cytometry was performed using separate staining panels rather than a single combined panel, including a Treg/myeloid panel and a CD8^+^ T-cell functional panel. Antibodies sharing the same fluorochrome were not used in the same staining tube. CD8^+^ and CD4^+^ T cells were quantified within live singlet CD45^+^ cells. Tregs were defined as CD45^+^CD4^+^FoxP3^+^ cells. Macrophages were defined as CD45^+^CD11b^+^F4/80^+^ cells using the anti-mouse CD11b clone M1/70 and anti-mouse F4/80 clone BM8. Myeloid-derived suppressor cells (MDSCs) were defined as CD45^+^CD11b^+^Gr-1^+^ cells using the anti-mouse CD11b clone M1/70 and anti-mouse Ly6G/Ly6C (Gr-1) clone RB6-8C5. For CD8^+^ T-cell functional profiling, IFN-γ was measured after 4 h of *ex vivo* PMA/ionomycin restimulation followed by intracellular cytokine staining. IFN-γ^+^, GZMB^+^, PRF1^+^, and Ki67^+^ values were reported within the live singlet CD8^+^ T-cell parent gate unless otherwise indicated. The complete antibody panel, including clone, fluorochrome, supplier, catalog number, and dilution, is provided in Table 1. Representative gating hierarchies for the separate panels are provided in Supplementary Figure S1.

### 2.8 Immunohistochemistry, multiplex immunofluorescence, and H-score quantification

Formalin-fixed, paraffin-embedded tumor sections were stained for IDO1, TDO2, or CD8 using commercially validated primary antibodies at optimized working dilutions. IHC was performed using the PV-9000 Universal Two-Step IHC Detection Kit according to the manufacturer’s protocol. Antibody clones, suppliers, catalog numbers, and working dilutions are listed in Table 1. IHC staining was quantified using the H-score method: H-score = 1 × (% weak staining) + 2 × (% moderate staining) + 3 × (% strong staining), with a total range of 0–300. H-score analysis was performed on at least five non-overlapping fields per section at ×20 magnification by investigators blinded to treatment allocation. Negative controls were generated by omitting the primary antibody. No separate positive-control tissue sections were processed in parallel.

For multiplex immunofluorescence, sections were stained with anti-CD3 and anti-α-SMA primary antibodies, followed by species-matched Alexa Fluor-conjugated secondary antibodies. Slides were mounted using ProLong Gold Antifade Mountant with DAPI. Quantification was performed on multiple non-overlapping fields by investigators blinded to the treatment group. Representative IHC and multiplex immunofluorescence images include scale bars in the revised figure files.

### 2.9 Statistical analysis

Data are presented as the mean ± SD unless otherwise specified. Two groups were compared using a two-tailed Student’s t-test or Wilcoxon rank-sum test as appropriate. Multiple groups were compared using one-way or two-way ANOVA followed by Tukey’s or Dunnett’s correction. Tumor growth curves were analyzed using two-way ANOVA with repeated measurements where applicable. Survival was analyzed using Kaplan–Meier estimates with the log-rank test. A *p*-value < 0.05 was considered statistically significant (*p < 0.05, **p < 0.01, and ***p < 0.001). Analyses were performed using GraphPad Prism and R.

**FIGURE 1:**
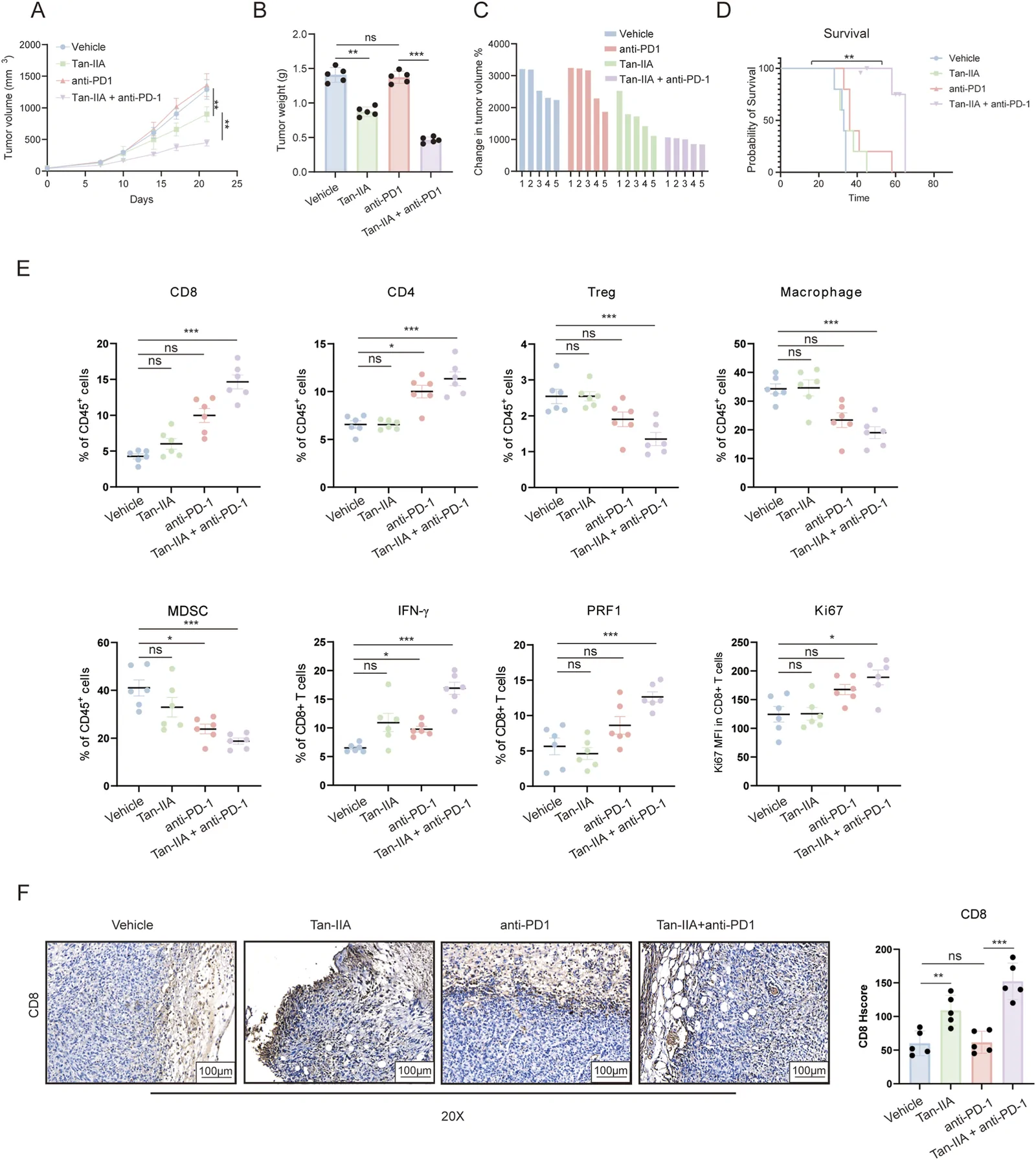
Tan-IIA sensitizes checkpoint-resistant 4T1 tumors to anti-PD-1 and remodels the immune microenvironment. **(A)** Tumor growth and **(B)** endpoint tumor weight across vehicle, Tan-IIA, anti-PD-1, and combination groups. **(C)** Per-animal waterfall plot showing percentage change in tumor volume from the treatment-start baseline to the endpoint on Day 21: [(tumor volume on Day 21 − tumor volume on Day 0)/tumor volume on Day 0] × 100. Each bar represents one mouse, and animals were sorted within each treatment group. **(D)** Kaplan–Meier survival analysis using humane endpoint criteria. In the revised survival analysis, anti-PD-1 monotherapy was not significantly different from the vehicle group, whereas Tan-IIA plus anti-PD-1 significantly prolonged survival compared with both the vehicle group and anti-PD-1 monotherapy. **(E)** Flow-cytometric immune profiling of CD8^+^ T cells, CD4^+^ T cells, FoxP3^+^ Tregs, macrophages, MDSCs, IFN-γ, PRF1, and Ki67. CD8^+^ T cells, CD4^+^ T cells, FoxP3^+^ Tregs, macrophages, and MDSCs are reported as percentages of live singlet CD45^+^ cells, whereas IFN-γ^+^, PRF1^+^, and Ki67^+^ populations are reported as percentages of live singlet CD8^+^ T cells. **(F)** Representative CD8 IHC and CD8 H-score. CD8 H-scores were quantified from at least five non-overlapping ×20 fields per section by blinded assessment. Scale bars, 100 µm. Body-weight curves during treatment are provided in Supplementary Figure S3. Data are presented as the mean ± SD; n = 5 mice per group unless otherwise stated. Tumor growth was analyzed using two-way ANOVA; survival was analyzed using the log-rank test; endpoint immune profiling used one-way ANOVA with multiple-comparison correction; ns, not significant; *p < 0.05, **p < 0.01, and ***p < 0.001.

Waterfall plots were generated using the percentage change in tumor volume from the treatment-start baseline to the Day 21 endpoint, as shown in Figure 1C: [(tumor volume on Day 21 − tumor volume on Day 0)/tumor volume on Day 0] × 100. Individual animals were plotted separately and sorted within each treatment group. Positive values indicate tumor growth relative to baseline, whereas negative values, if present, indicate tumor shrinkage relative to baseline. Unless otherwise stated, ns denotes not significant.

## 3 Results

### 3.1 Network pharmacology and TCGA analysis nominate IDO1/TDO2–kynurenine metabolism as a Tan-IIA-associated axis of CD8 dysfunction

**FIGURE 2:**
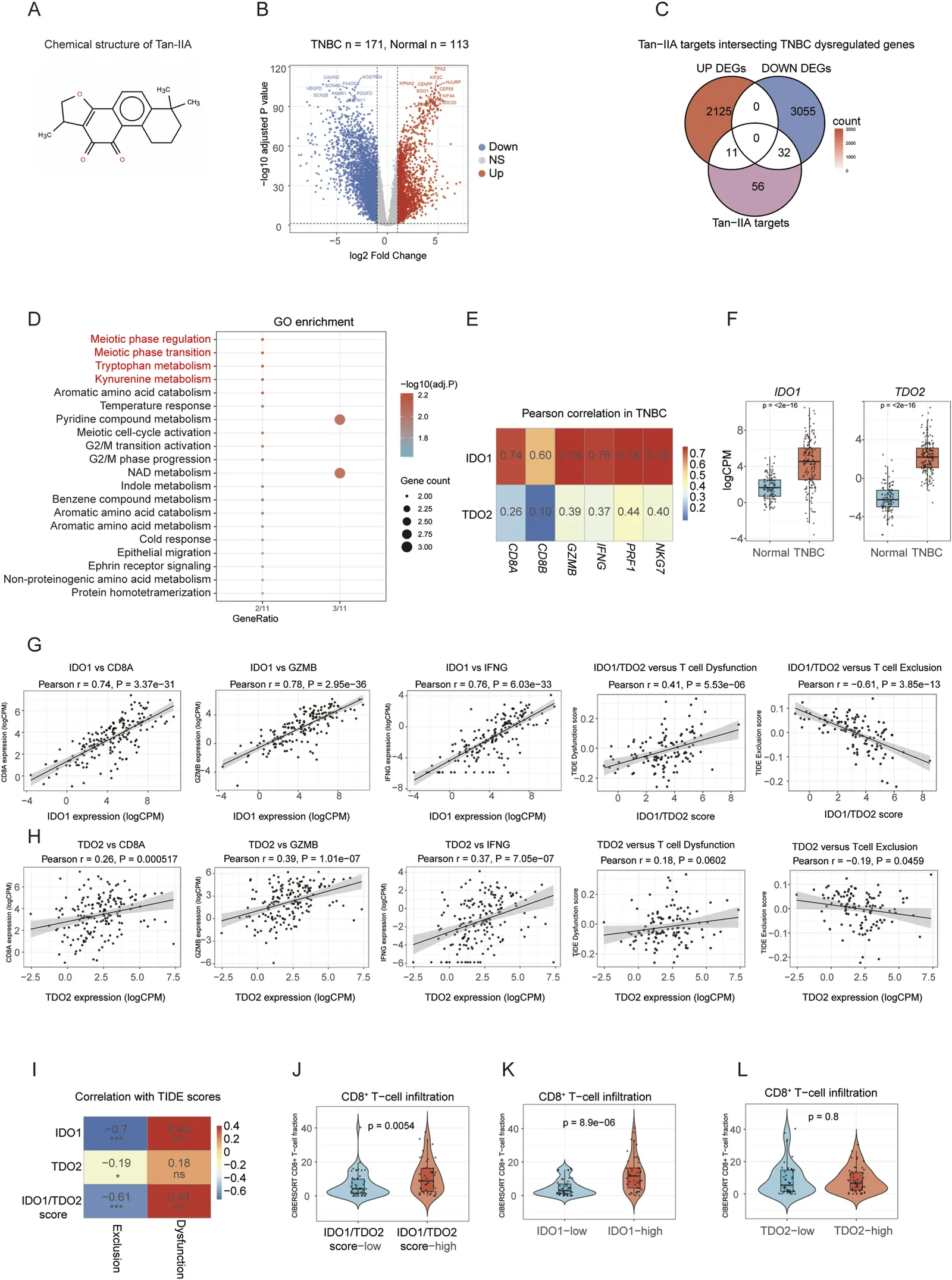
Network pharmacology and TCGA–BRCA analysis identify the IDO1/TDO2–kynurenine axis in TNBC. **(A)** Chemical structure of Tan-IIA. **(B)** Volcano plot of TNBC versus normal differential expression (TNBC n = 171; normal n = 113). **(C)** Venn diagram of predicted Tan-IIA-associated targets intersecting TNBC up- and downregulated genes. **(D)** GO/KEGG enrichment of overlapping genes. **(E)** Pearson correlation heatmap between IDO1/TDO2 and cytotoxic markers in TCGA–BRCA TNBC samples. **(F)** IDO1 and TDO2 expression in normal versus TNBC tissue. **(G,H)** Representative Pearson correlations of IDO1 or TDO2 with cytotoxic markers and TIDE scores. **(I)** Summary heatmap of correlations with TIDE dysfunction and exclusion. **(J–L)** CIBERSORT CD8^+^ T-cell fractions stratified by IDO1/TDO2 score, IDO1, and TDO2. The IDO1/TDO2 composite score was calculated in TCGA–BRCA TNBC samples as the mean of separately z-scored IDO1 and TDO2 logCPM values. Its positive association with TIDE dysfunction and negative association with TIDE exclusion support an inflamed-yet-dysfunctional immune phenotype rather than an immune-excluded phenotype. Pearson correlation was used for continuous variables, and Wilcoxon rank-sum tests were used for two-group comparisons; ns, not significant; *p < 0.05, **p < 0.01, and ***p < 0.001.

The chemical structure of Tan-IIA is shown in Figure 2A. Differential expression analysis of the TCGA–BRCA cohort (TNBC, n = 171; normal, n = 113) identified a large set of dysregulated genes (Figure 2B). Intersecting predicted Tan-IIA-associated targets with the TNBC up- and downregulated genes yielded 43 shared genes, including 11 overlapping with upregulated genes and 32 overlapping with downregulated genes (Figure 2C; Supplementary Table S1). Functional enrichment of the overlap highlighted tryptophan metabolism, kynurenine metabolism, and aromatic amino-acid catabolism (Figure 2D), and IDO1 and TDO2, the rate-limiting enzymes in kynurenine production, were present among the upregulated overlapping genes. Consistent with this, IDO1 and TDO2 were overexpressed in TNBC relative to normal tissue (both *p* < 2 × 10^−16^; Figure 2F).

To place these enzymes in an immunological context, we correlated their expression with cytotoxic markers in TNBC samples. IDO1 was strongly and positively correlated with CD8A, CD8B, GZMB, IFNG, PRF1, and NKG7 (Pearson r = 0.60–0.78), whereas TDO2 showed weaker associations (r = 0.10–0.44) (Figures 2E,G,H). This positive coupling of IDO1 to a cytotoxic signature is consistent with IFN-γ-driven adaptive immune resistance rather than simple immune desertion. Accordingly, the IDO1/TDO2 score correlated positively with the TIDE dysfunction score (r = 0.41, *p* = 5.5 × 10^−6^) but negatively with the TIDE exclusion score (r = −0.61, *p* = 3.9 × 10^−13^; Figure 2I; Supplementary Table S3). This pattern indicates that high IDO1/TDO2 activity is associated with impaired T-cell function despite the presence of immune cells, rather than with the physical exclusion or absence of T cells. Thus, the IDO1/TDO2 score marked an inflamed-yet-dysfunctional immune state in TNBC. IDO1-high tumors displayed greater CIBERSORT CD8^+^ T-cell fractions (*p* = 8.9 × 10^−6^), as did IDO1/TDO2-score-high tumors (*p* = 0.0054), whereas TDO2 alone showed no significant relationship (*p* = 0.8) (Figures 2J–L; Supplementary Table S4). Together, these data indicate that IDO1/TDO2 mark an inflamed-yet-suppressed state in which cytotoxic cells are present but functionally restrained.

### 3.2 Tan-IIA inhibits TNBC cell growth and suppresses IDO1/TDO2 expression

**FIGURE 3:**
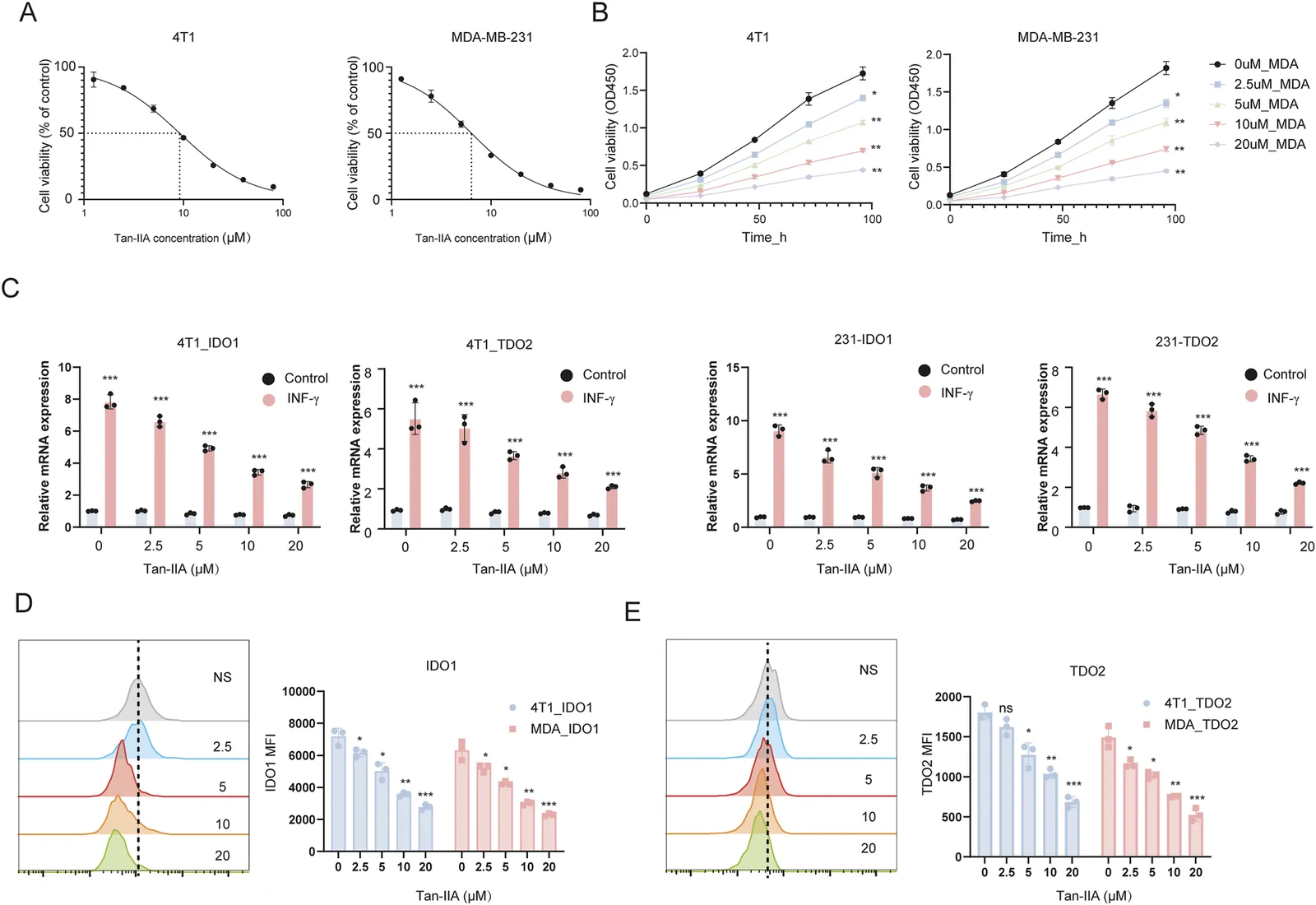
Tan-IIA inhibits TNBC growth and suppresses IDO1/TDO2. **(A)** Dose–response viability curves and IC50 values in 4T1 and MDA-MB-231 cells at 72 h. **(B)** Time-course viability at 0–20 µM Tan-IIA. **(C)** RT-qPCR analysis of mouse *Ido1/Tdo2* and human IDO1/TDO2 under species-matched IFN-γ stimulation across Tan-IIA doses. **(D)** Flow-cytometric IDO1 MFI with representative histograms. **(E)** Flow-cytometric TDO2 MFI with representative histograms. Comparisons in dose–response panels used the IFN-γ-stimulated or vehicle-treated reference group indicated in the panel. Data are presented as the mean ± SD from three independent biological experiments (n = 3). One-way ANOVA with Dunnett’s or Tukey’s multiple-comparison correction was used for multi-dose comparisons, and two-way ANOVA was used for time-course analysis; ns or NS, not significant; *p < 0.05, **p < 0.01, and ***p < 0.001.

Tan-IIA reduced the viability of both 4T1 and MDA-MB-231 cells in a dose-dependent manner, with 72-h IC_50_ values of approximately 9.2 µM and 6.1 µM, respectively (Figure 3A), and progressively inhibited growth over 0–96 h at 2.5–20 µM (Figure 3B). Sub-cytotoxic to moderate doses (0–20 µM) were therefore used for mechanistic experiments. Species-matched IFN-γ robustly induced IDO1/Ido1 and TDO2/Tdo2 transcripts in both lines, and Tan-IIA suppressed this induction dose-dependently (Figure 3C), with the strongest effect at 10–20 µM. At the protein level, intracellular flow cytometry showed that Tan-IIA progressively lowered IDO1 and TDO2 MFI in both 4T1 and MDA-MB-231 cells (Figures 3D,E). Thus, Tan-IIA antagonizes the inflammatory induction of both rate-limiting enzymes in the kynurenine pathway.

### 3.3 Tan-IIA restores CD8^+^ T-cell function by lowering kynurenine and reducing AhR target-gene expression

**FIGURE 4:**
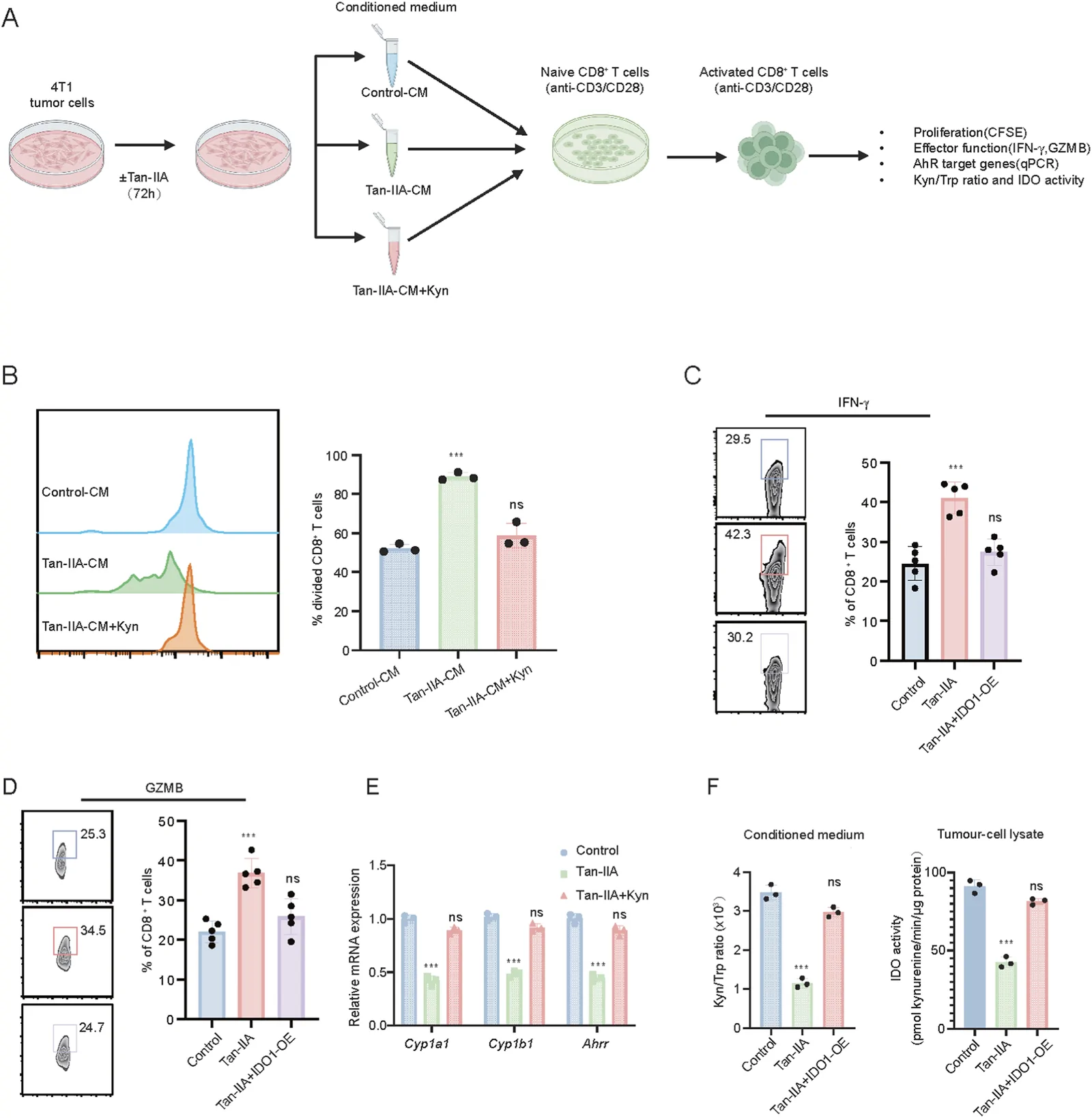
Tan-IIA restores CD8^+^ T-cell function via the kynurenine–AhR pathway. **(A)** Co-culture schematic using tumor-cell-conditioned medium with or without Tan-IIA and kynurenine add-back. Figure 4A was created by the authors using licensed BioRender content and was not reproduced or adapted from a previously published figure. Citation and source: Created in BioRender. Wu, Y. (2026) https://BioRender.com/hppgl5l. **(B)** CFSE-based CD8^+^ T-cell proliferation under control-CM, Tan-IIA-CM, and Tan-IIA-CM + Kyn conditions. **(C,D)** Representative flow-cytometry plots and quantification of IFN-γ^+^ and GZMB^+^ cells among live singlet CD8^+^ T cells, including IDO1-OE rescue. Displayed gate values represent the percentage of cytokine-positive cells within the CD8^+^ parent gate. **(E)** AhR target genes Cyp1a1, Cyp1b1, and Ahrr in CD8^+^ T cells. **(F)** The Kyn/Trp ratio measured in tumor-cell-conditioned medium and IDO enzymatic activity measured in tumor-cell lysates, with IDO1-OE rescue. Kyn/Trp is reported as Kyn/Trp × 10^3^, and IDO activity is reported as pmol kynurenine generated per min per µg protein. IDO1-OE validation is provided in Supplementary Figures S2A,B. Data are presented as the mean ± SD from three independent biological experiments (n = 3). One-way ANOVA with Tukey’s correction was used; ns, not significant; ***p < 0.001.

To assess functional consequences, activated CD8^+^ T cells were exposed to tumor-conditioned medium (Figure 4A). Tan-IIA-CM increased the fraction of divided CD8^+^ T cells relative to control-CM, and this increase was reversed by exogenous kynurenine add-back (Figure 4B). IDO1 overexpression used in the rescue experiments was confirmed by RT-qPCR and immunoblotting (Supplementary Figures S2A,B). Tan-IIA-CM also increased the proportions of IFN-γ^+^ and granzyme B^+^ CD8^+^ T cells; IDO1 overexpression in tumor cells substantially attenuated these gains (Figures 4C,D). Mechanistically, Tan-IIA-CM downregulated the AhR target genes *Cyp1a1*, *Cyp1b1*, and *Ahrr* in CD8^+^ T cells, and kynurenine supplementation restored their expression (Figure 4E). Tan-IIA also reduced the Kyn/Trp ratio in tumor-cell-conditioned medium and decreased IDO enzymatic activity in tumor-cell lysates, both of which were substantially reversed by IDO1 overexpression (Figure 4F). These complementary metabolite add-back and IDO1 rescue experiments support a model in which Tan-IIA relieves CD8^+^ T-cell suppression through the IDO1–kynurenine–AhR pathway rather than through a non-specific effect of conditioned medium.

### 3.4 Tan-IIA suppresses tumor IDO1/TDO2, increases T-cell infiltration, and shows CD8-dependent antitumor activity

**FIGURE 5:**
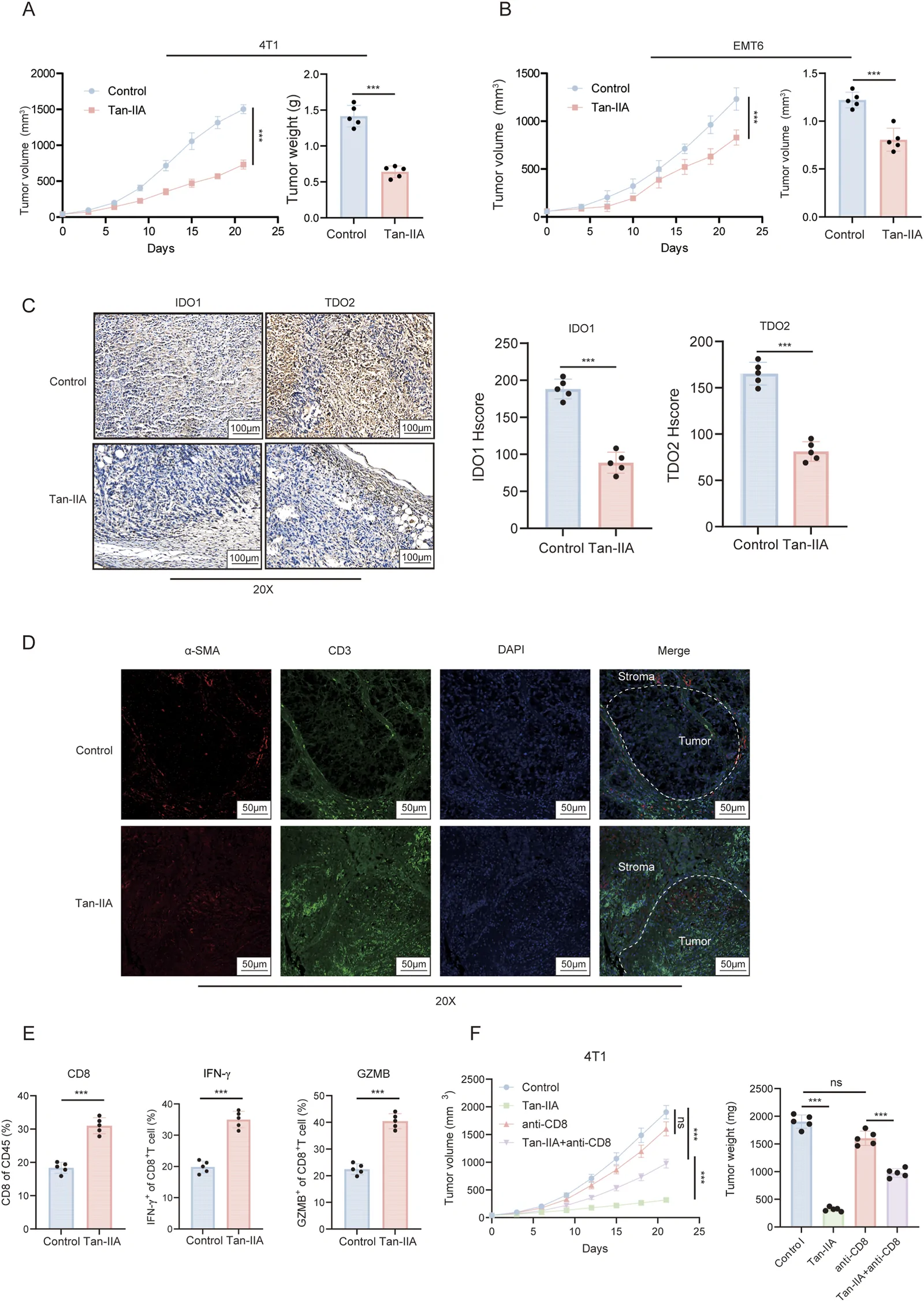
Tan-IIA suppresses tumor IDO1/TDO2 and enhances T-cell infiltration, and its antitumor efficacy is CD8-dependent. **(A,B)** Tumor growth and end-point tumor weight in 4T1 and EMT6 syngeneic mammary tumor models treated with vehicle or Tan-IIA. **(C)** Representative IDO1 and TDO2 IHC and H-score quantification. H-scores were calculated from at least five non-overlapping 20× fields per section by blinded assessment using the following formula: H-score = 1 × (% weak staining) + 2 × (% moderate staining) + 3 × (% strong staining). Scale bars, 100 µm. **(D)** Multiplex IF of α-SMA, CD3, and DAPI showing T-cell distribution relative to stromal and tumor regions. Scale bars, 50 µm. **(E)** Flow-cytometric quantification of intratumoral CD8^+^ T cells and IFN-γ^+^ or GZMB^+^ CD8^+^ T cells. **(F)** CD8-depletion experiment showing that anti-CD8 treatment blunts the antitumor effect of Tan-IIA. Data are presented as the mean ± SD; n = 5 mice per group unless otherwise stated. Tumor growth was analyzed using two-way ANOVA; endpoint comparisons used two-tailed t-tests or one-way ANOVA as appropriate; ns, not significant; ***p < 0.001.

Tan-IIA monotherapy was next evaluated in immunocompetent syngeneic mammary tumor models. In 4T1-bearing mice, Tan-IIA slowed tumor growth and reduced endpoint tumor weight (Figure 5A). A similar direction of tumor-growth inhibition was observed in the EMT6 validation model (Figure 5B). IHC analysis of 4T1 tumors showed that Tan-IIA reduced intratumoral IDO1 and TDO2 H-scores (Figure 5C), consistent with the *in vitro* suppression of the kynurenine pathway. Multiplex immunofluorescence further showed reduced stromal restriction and increased CD3^+^ T-cell distribution within tumor regions after Tan-IIA treatment (Figure 5D). Flow-cytometric profiling confirmed increased CD8^+^ T-cell infiltration and higher IFN-γ+ and GZMB+ fractions among tumor-infiltrating CD8^+^ T cells (Figure 5). Finally, antibody-mediated CD8 depletion substantially blunted the antitumor effect of Tan-IIA (Figure 5F), indicating that cytotoxic T cells contribute materially to Tan-IIA efficacy *in vivo*.

### 3.5 Tan-IIA sensitizes checkpoint-resistant 4T1 tumors to anti-PD-1 and remodels the immune microenvironment

Because relieving metabolic suppression should enhance checkpoint responsiveness, we combined Tan-IIA with anti-PD-1 in the 4T1 model. Consistent with the checkpoint-resistant nature of 4T1 tumors, anti-PD-1 monotherapy showed minimal tumor control and closely resembled the vehicle group. Tan-IIA monotherapy moderately delayed tumor growth, whereas Tan-IIA combined with anti-PD-1 produced the strongest suppression of tumor volume and endpoint tumor weight (Figures 1A,B). The per-animal waterfall plot showed the same pattern, with the combination group exhibiting the smallest increase in tumor volume from the treatment-start baseline to the endpoint (Figure 1C). Kaplan–Meier analysis showed that anti-PD-1 monotherapy did not significantly prolong survival relative to vehicle, whereas Tan-IIA combined with anti-PD-1 produced the greatest survival benefit (Figure 1D).

Immune profiling explained the improved therapeutic response (Figure 1E). Anti-PD-1 monotherapy alone produced little change in CD8^+^ T-cell infiltration, effector function, or suppressive myeloid/Treg populations relative to vehicle. Tan-IIA increased intratumoral CD8^+^ and CD4^+^ T-cell frequencies and reduced suppressive populations, whereas the combination produced the most pronounced shift toward an effector-dominated microenvironment, with higher IFN-γ, PRF1, and Ki67 in CD8^+^ T cells and lower FoxP3^+^ Treg, macrophage, and MDSC content. CD8 immunohistochemistry confirmed the greatest cytotoxic infiltration in combination-treated tumors, as reflected by the highest CD8 H-score (Figure 1F). Body-weight curves for all treatment groups are provided in Supplementary Figure S3 and showed no evident treatment-associated body-weight loss, consistent with acceptable systemic tolerability. Collectively, Tan-IIA converts anti-PD-1-resistant 4T1 tumors into a more responsive state by relieving IDO1/TDO2–kynurenine-mediated CD8^+^ T-cell dysfunction.

## 4 Discussion

In this study, we identify tumor tryptophan catabolism as a mechanism through which Tan-IIA reshapes the TNBC immune microenvironment. Starting from the intersection of predicted Tan-IIA-associated targets and TNBC transcriptomic changes, we identified IDO1, TDO2, and the kynurenine pathway and then validated experimentally that Tan-IIA suppresses these enzymes, lowers the Kyn/Trp ratio, reduces AhR target-gene expression, and restores CD8^+^ T-cell function. The functional reversal by exogenous kynurenine and by tumor-cell IDO1 overexpression is central because it links the immune benefit to the IDO1–kynurenine–AhR axis rather than to a non-specific conditioned-medium effect.

An important nuance is that IDO1 expression correlated positively, not negatively, with cytotoxic markers and CD8^+^ infiltration in TNBC. This is consistent with IDO1 being an IFN-γ-inducible adaptive resistance node: T-cell-derived IFN-γ induces IDO1, whose product, kynurenine, then restrains the very cells that triggered it (Munn and Mellor, 2016; Spranger et al., 2013). Such tumors are better described as inflamed but dysfunctional rather than simply immune-deserted or excluded. The positive association of the IDO1/TDO2 score with TIDE dysfunction and its negative association with TIDE exclusion support this interpretation. Relieving this metabolic brake is mechanistically rational and may explain why Tan-IIA produced its greatest effects in combination with anti-PD-1.

Our findings also relate to the clinical disappointment of selective IDO1 inhibition, exemplified by the ECHO-301/KEYNOTE-252 trial, in which epacadostat failed to improve outcomes over pembrolizumab alone (Long et al., 2019). A possible contributor is redundancy within tryptophan catabolism, including TDO2-mediated kynurenine production (Platten et al., 2019; Opitz et al., 2011). By suppressing both IDO1 and TDO2 expression in tumor cells, Tan-IIA may reduce compensatory kynurenine generation; however, this remains an inference that requires direct testing with selective genetic and pharmacological perturbations of each enzyme. The current data establish IDO1-dependent rescue and support broader IDO1/TDO2 pathway involvement, but they do not prove that dual-enzyme suppression is the only mechanism of action.

From a translational standpoint, Tan-IIA is attractive because it combines direct anti-proliferative activity against TNBC cells (Wang et al., 2005; Fang et al., 2021; He et al., 2023; Lin et al., 2013) with immunometabolic remodeling. The CD8-depletion experiment indicates that immune relief, not cytotoxicity alone, contributes to the *in vivo* benefit. In the 4T1 model, anti-PD-1 monotherapy was weak, as expected for a poorly checkpoint-responsive tumor, whereas Tan-IIA sensitized tumors to anti-PD-1 and produced stronger tumor control, longer survival, and higher CD8 infiltration. The revised Kaplan–Meier analysis is consistent with the tumor-volume data: anti-PD-1 monotherapy did not show a significant survival advantage over the vehicle group, whereas the combination treatment produced the greatest survival benefit. These findings support Tan-IIA as a candidate metabolic adjuvant for ICB-resistant TNBC.

Several limitations should be acknowledged. The bioinformatic associations are correlative and derived from bulk transcriptomics; single-cell or spatial analyses would better resolve which cell compartments express IDO1/TDO2 and respond to Tan-IIA. The upstream molecular event by which Tan-IIA suppresses IDO1/TDO2 transcription, such as interference with IFN-γ/JAK–STAT1 signaling or another route, remains to be defined. Although IDO1 overexpression partially rescued the phenotype, complementary IDO1 knockdown and TDO2- and AhR-specific studies would strengthen causality. *In vivo* studies focused mainly on syngeneic murine models; patient-derived organoids, humanized immune models, or clinical tissue validation will be needed before translation. Despite these limitations, the data provide a coherent mechanistic case that Tan-IIA relieves IDO1/TDO2–kynurenine–AhR metabolic immunosuppression and sensitizes TNBC to anti-PD-1.

## 5 Conclusion

Tan-IIA targets the IDO1/TDO2–kynurenine–AhR axis to relieve metabolic suppression of CD8^+^ T cells and remodel the TNBC microenvironment toward an effector-dominated state. In combination with anti-PD-1, it produces superior tumor control and survival in the checkpoint-resistant 4T1 model. These results support further development of Tan-IIA as a microenvironment-remodeling partner to overcome resistance to immune checkpoint blockade in triple-negative breast cancer.

## Data Availability

The datasets presented in this study can be found in online repositories. The names of the repository/repositories and accession number(s) can be found in the article/Supplementary Material.
